# Patch Testing in Paediatric Drug Hypersensitivity Reactions: Current Evidence and Clinical Implications

**DOI:** 10.1111/cod.70203

**Published:** 2026-06-10

**Authors:** Gabriele Simonetti, Francesco Catamerò, Giulia Liccioli, Leonardo Tomei, Simona Barni, Mattia Giovannini, Lucrezia Sarti, Francesca Mori

**Affiliations:** ^1^ Department of Health Sciences University of Florence Florence Italy; ^2^ Allergy Unit Meyer Children's Hospital IRCCS Florence Italy

**Keywords:** children, diagnosis, drug allergy, patch test, SCARs

## Abstract

Drug hypersensitivity reactions (DHRs) are common in children and are diagnostically challenging, with cutaneous manifestations being the most common presentation. Patch testing (PT) is a well‐established tool for investigating delayed T‐cell‐mediated reactions and is widely used in patients with contact dermatitis. However, its role in paediatric DHRs remains unclear. Available data indicate that PT is safe and particularly useful when drug provocation testing is contraindicated, such as in patients with severe cutaneous adverse reactions. However, diagnostic yield varies considerably among drug classes and clinical phenotypes. Sensitivity is generally low for beta‐lactam and most non–beta‐lactam antibiotics, which limits their standalone value in common exanthematous reactions. Higher positivity rates have been reported for antiepileptic drugs, and PT may help identify the culprit drug and assess its cross‐reactivity. However, evidence supporting the use of nonsteroidal anti‐inflammatory drugs and paracetamol remains scarce. Methodological heterogeneity and limited paediatric‐specific data further constrain this interpretation. Overall, PT should be considered as a complementary diagnostic tool integrated with clinical history and other tests, highlighting the need for standardized protocols and prospective paediatric studies.

## Key Message

Patch testing is a safe but variably sensitive complementary tool for diagnosing delayed paediatric drug hypersensitivity, with the highest utility for antiepileptic reactions and limited value for most antibiotics.

## 1 Introduction

Drug hypersensitivity reactions (DHRs) in children represent a clinically heterogeneous group of adverse events, ranging from benign exanthems to severe and potentially life‐threatening syndromes [1, 2]. Up to 10% of parents report suspected DHRs in their children, although only a minority of these cases are confirmed as true allergic reactions after a comprehensive evaluation [2]. Beta‐lactam antibiotics, antiepileptic drugs (AEDs), and nonsteroidal anti‐inflammatory drugs (NSAIDs) are frequently involved in paediatric DHRs [1, 2]. Cutaneous adverse drug reactions (CADRs) are among the most common manifestations of drug hypersensitivity in children, with prevalence estimates ranging from 2% to 10% depending on the population and type of drug exposure [3]. Notably, most CADRs in children are delayed reactions typically mediated by T‐cell‐dependent (type IV) immune mechanisms. Clinically, CADRs encompass a wide spectrum, from benign exanthems to severe cutaneous adverse reactions (SCARs), including Stevens‐Johnson syndrome (SJS), toxic epidermal necrolysis (TEN), drug reaction with eosinophilia and systemic symptoms (DRESS), and acute generalized exanthematous pustulosis (AGEP) [2, 4].

A major challenge in the paediatric population is the frequent coexistence of viral or bacterial infections and drug exposure, which complicates the interpretation of cutaneous eruptions [5, 6]. Many rashes occurring during antibiotic therapy, particularly with beta‐lactams, are non‐immunological and infection‐related, leading to a substantial overdiagnosis of drug allergies [2, 7]. Accurate identification of the culprit drug is essential to prevent recurrence and avoid mislabelling children as drug‐allergic; however, this is often challenging owing to polypharmacy and overlapping clinical features [8, 9].

Patch testing (PT) is an established diagnostic tool for investigating delayed (type IV) hypersensitivity reactions and is widely used in allergic patients with contact dermatitis [1, 10, 11, 12, 13]. In the context of DHRs, PT is considered a first‐line skin test for SCARs because of its favourable safety profile and low risk of systemic reactions or disease reactivation [1]. Current recommendations support the use of PT for risk stratification and identification of causative drugs in severe delayed reactions, such as DRESS, AGEP, and fixed drug eruption (FDE), while acknowledging the limited sensitivity of SJS/TEN to certain drug classes [1, 14]. The number of suspected drugs also plays a crucial role; in patients exposed to multiple drugs, identification of the culprit agent is particularly challenging, and PT results must be interpreted cautiously within the overall clinical context [15, 16]. Moreover, higher PT positivity has been reported with AEDs, whereas lower rates have been observed with antibiotics, with the diagnostic yield influenced by the drug class and reaction phenotype [9, 12]. A negative PT result does not exclude drug causality, necessitating integration with the clinical history and, when appropriate, drug provocation testing [1].

In children, the technical aspects of PT require adaptation according to age, body surface area, and patient compliance. Additionally, available safety data indicate that PT is well‐tolerated in adult and paediatric populations, with adverse events being rare and typically limited to mild and localized reactions [14, 17]. Although consensus documents provide recommendations on allergen concentrations, vehicles, and reading times, practice variability persists across centres, and paediatric‐specific standardized protocols for drug PT remain limited [18, 19].

This narrative review aims to examine the available literature on the use of PT in paediatric DHRs, focusing on clinical indications, diagnostic yields across different reaction phenotypes and drug classes, methodological considerations, and existing gaps in paediatric data.

A narrative literature search was conducted in PubMed using combinations of the following keywords: (“patch test” OR “patch testing”) AND (“drug allergy” OR “drug hypersensitivity reactions”) AND (“children” OR “paediatrics”). The search was performed until February 2026, and only articles published in English were selected. The articles were selected based on their direct relevance to the clinical use of PT in children and adolescents with DHRs. The inclusion criteria prioritised original research, particularly large observational real‐world studies, as well as review articles or guideline documents that provided a critical context. Case reports were included when they offered unique insights not available in larger studies. Articles that did not focus on paediatric populations or did not discuss PT in DHRs were excluded.

## 2 PT Methodology in Paediatric DHRs

PT is a skin‐based diagnostic procedure used to investigate type IV hypersensitivity reactions by assessing T‐cell‐mediated responses to suspected drugs applied epicutaneously under occlusion [12]. The procedure involves applying the suspected drug, typically at concentrations of 10% and 30% in petrolatum or water, under occlusion to the skin, most commonly on the back, for 48 h, followed by serial readings at 48, 72, and 96 h to detect reactions indicative of delayed hypersensitivity [8, 12].

The PT results are interpreted according to the International Contact Dermatitis Research Group criteria, as described by Johansen et al., based on the morphological assessment of skin reactions (erythema, infiltration, papules, and vesicles) through inspection and palpation, with positive responses defined by the presence of + to +++ reactions, indicating an allergic reaction [20, 21].

Despite this immunological rationale, the integration of PT into routine paediatric drug allergy work‐up remains complex because several methodological and patient‐related factors influence test performance, particularly in children. Paediatric skin characteristics, including increased permeability, may predispose children to irritant reactions, whereas limited tolerance to prolonged occlusion and difficulties in interpreting faint or borderline responses further complicate response assessment [17]. However, the methodological variability remains a major challenge. For most drugs, optimal testing concentrations and vehicles have not been fully validated, and standardized preparations are often unavailable. Many agents require preparation by crushing tablets or opening capsules and suspending the active ingredient in an appropriate vehicle, with attention paid to the excipients to minimize the risk of false‐negative or irritant reactions. These issues contribute to the wide range of sensitivities and specificities reported in the literature, both in paediatric cohorts and in studies including mixed‐age populations [18].

The timing of the reaction also affects performance. Expert consensus generally recommends testing 2–6 months after the complete resolution of the eruption, particularly for severe reactions such as DRESS or AGEP, to minimize false negatives and ensure immunological recovery [1, 12]. Moreover, patients should be evaluated for at least 4 weeks after discontinuation of systemic steroids (> 10 mg prednisone equivalent) or other immunosuppressants.

PT is considered safe with exceedingly rare systemic reactions, making it a valuable option when drug provocation is contraindicated [22]. Within this framework, PT is used selectively as a complementary tool for confirming delayed hypersensitivity when clinical suspicion is strong and when a drug provocation test, despite being the gold standard, cannot be safely performed.

## 3 Evidence for PT in Paediatric Population Across Drug Classes

The DHRs to multiple drug classes have been investigated to assess the diagnostic utility of PT, particularly in relation to beta‐lactam antibiotics, other antimicrobial agents, NSAIDs, and AEDs. As mentioned previously, these drug categories are among the most frequently prescribed and are disproportionately associated with DHRs, underscoring the need to clearly define the diagnostic role and clinical value of PT within this context. Table 1 summarizes the relevant studies available in the literature on the use of PT in the paediatric population, providing an overview of the study designs, drug classes investigated, and main diagnostic outcomes.

**TABLE 1:** Summary of main studies (not case reports) evaluating patch testing for drug hypersensitivity reactions in the paediatric population.

Study	Design and population	Drug classes	Reaction types	Main findings
Blanca‐López et al. (2009)	Prospective study; 20 children (mean age 4.9 years) with confirmed non‐immediate aminopenicillin hypersensitivity	Aminopenicillins	MPE, delayed urticaria, serum sickness‐like	PT and IDT showed low diagnostic yield: only 1/20 confirmed cases had positive PT and IDT. 19/20 were diagnosed by positive drug provocation test. No adverse reactions reported to PT.
Atanaskovic‐Markovic et al. (2016)	Prospective study; 1026 children (mean age 7.7 years) with histories of nonimmediate reactions to beta‐lactams	Beta‐lactams	Mainly MPE and delayed urticaria/angioedema; 2 SJS	Only 76/1026 (7.4%) children had confirmed non‐immediate beta‐lactam hypersensitivity. 57/1026 (5.5%) had positive IDT, and 18 of these also had positive PT. 19 additional cases were confirmed only by DPT. No adverse reactions reported to PT.
Atanaskovic‐Markovic et al. (2019)	Prospective study; 100 children (mean age 8.67 years) with suspected hypersensitivity to AEDs	AEDs	Mainly MPE, delayed urticaria, and SCARs	Hypersensitivity was confirmed in 66/100 (66%) children. PT showed high diagnostic yield, with 59/100 positive results. Seven children were positive to IDT. No adverse reactions reported to PT.
Liccioli et al. (2020)	Retrospective study; 54 children (median age 6.5 years) with SCARs	Antibiotics, AEDs, NSAIDs, terbinafine, and others	SCARs: DRESS, SJS/TEN, AGEP, plus rare bullous reactions	PT positivity was limited and varied by drug class and reaction type. In DRESS, positivity was observed with beta‐lactams (1/5), non–beta‐lactam antibiotics (1/4), and antiepileptics (2/7). All PTs performed in SJS/TEN (beta‐lactams, non–beta‐lactams, and NSAIDs) were negative. Isolated positivity was observed in AGEP to hydroxychloroquine. No adverse reactions reported to PT.
Büyük Yaytokgil et al. (2021)	Retrospective study; 71 children (median age 7 years), 105 PTs for delayed CADRs or SCARs	Mainly AEDs and antibiotics	MPE, FDE, DRESS, SJS, AGEP	Overall PT positivity was 20/105 (28%). Positivity was higher for AEDs (18/44; 40.9%) than for antibiotics (2/48; 4.1%). Positive results were found in both mild and severe reactions. No adverse reactions reported to PT.
Costa Carvalho et al. (2022)	Retrospective study; 22 children (median age 13.5 years) with suspected CADRs undergoing PT	Antibiotics, AEDs, and others	MPE, DRESS, SJS/TEN	PT overall positivity was 4/22 (18%): in 2 of 17 (12%) MPE (to vancomycin and flucloxacillin), and in 2 of 5 (40%) SCARs (to carbamazepine in DRESS induced by oxcarbazepine and to ethosuximide in SJS/TEN overlap). Negative PT in benign beta‐lactam MPE were followed by tolerated re‐exposure/challenge in reported cases No adverse reactions reported to PT

Abbreviations: AEDs, antiepileptic drug; AGEP, acute generalized exanthematous pustulosis; CADR, cutaneous adverse drug reaction; DRESS, drug reaction with eosinophilia and systemic symptoms; FDE, fixed drug eruption; IDT, intradermal test; MPE, maculopapular exanthema; NSAIDs, nonsteroidal anti‐inflammatory drugs; PT, patch test; SCAR, severe cutaneous adverse reaction; SJS, Stevens‐Johnson syndrome; TEN, toxic epidermal necrolysis.

### 3.1 Beta‐Lactam Antibiotics

Beta‐lactam antibiotics, including penicillins and cephalosporins, are the most frequently prescribed classes of antibiotics in paediatric practice and are among the most commonly implicated in DHRs [17]. The spectrum of hypersensitivity reactions to beta‐lactams ranges from immediate IgE‐mediated reactions (urticaria, angioedema, and anaphylaxis) to delayed T‐cell‐mediated manifestations (maculopapular exanthema, FDE, and rarely, SCARs) [23]. The diagnostic evaluation of suspected beta‐lactam hypersensitivity in children has advanced substantially owing to international guidelines; however, the specific role of PT remains relatively limited compared with intradermal testing and drug provocation testing.

Evidence on the diagnostic utility of PT for non‐immediate DHRs to beta‐lactams in children suggests a consistently low sensitivity in routine clinical practice, although studies specifically focusing on the paediatric population remain limited [24]. In a multicentre retrospective study evaluating 872 patients aged > 15 years with non‐immediate reactions to penicillins, PT positivity varied according to clinical phenotype [24]. In patients with uncomplicated morbilliform drug eruptions, PT positivity increased markedly with eruption duration, ranging from 10.9% in reactions lasting 2–4 days to 82.7% in episodes lasting more than 28 days. Overall, PT and/or delayed‐reading intradermal tests were positive in 33.4% of the patients, whereas drug provocation tests were positive in only 3%.

In a large prospective cohort of 1026 children with suspected non‐immediate mild reactions to beta‐lactam antibiotics, primarily aminopenicillins (amoxicillin and ampicillin), benzylpenicillin, and several cephalosporins (e.g., ceftriaxone, cefixime, and cefprozil), a complete allergy workup was performed, including PT, intradermal tests, and drug provocation tests. Overall, only 76 of the 1026 children (7.4%) were confirmed to have non‐immediate hypersensitivity. Although delayed‐reading intradermal tests were positive in 57/76 confirmed cases, PTs were positive in only 18 children (1.7% of the total cohort) and were always associated with other positive tests. Most cases of PTs positivity involved aminopenicillins, particularly amoxicillin [25].

Data from a retrospective paediatric series further support this limited test yield. Across studies evaluating suspected DHRs, ranging from mild or moderate reactions to SCARs, positivity rates for beta‐lactam PT ranged from 0% to approximately 5%, with amoxicillin–clavulanate being most frequently implicated when positive results occurred [17, 26, 27]. In SCARs, including DRESS and SJS/TEN, PT demonstrated inconsistent performance; occasional positivity was observed in DRESS, whereas all PTs performed in SJS/TEN were negative. These findings suggest that although PT may occasionally contribute to the etiological clarification of selected SCAR phenotypes, particularly DRESS, its overall sensitivity remains modest [4]. Data on the sensitivity rates of the PT in FDE are limited. A single isolated positivity in a 4‐year‐old child with FDE to amoxicillin–clavulanate has been reported [17].

Moreover, in adult cohorts, multiple drug reactivity, as demonstrated by positive PT for different drug classes administered during the acute phase of DRESS, is relatively frequent and far more common than in other SCARs. This phenomenon likely reflects virus‐ and inflammation‐driven immune activation, whereby costimulatory signals enhance sensitization to multiple concomitant drugs. Such sensitization may persist over the long term, with positive PTs documented years after the initial DRESS episode [14].

Higher PT positivity to beta‐lactams has been described in selected contexts, such as in patients with prior DRESS induced by non‐antibiotic drugs who were subsequently exposed to antibiotics, where sensitization rates appeared unexpectedly elevated and cross‐reactivity among penicillins and cephalosporins was documented [28]. Although these observations raise questions about immune dysregulation and secondary sensitization, the small sample sizes and retrospective design warrant cautious interpretation of the results.

Moreover, a single paediatric case of ceftriaxone‐induced AGEP has been reported, with positive PT confirming the diagnosis [29].

Finally, transient, delayed hypersensitivity reactions, particularly to aminopenicillins, are commonly observed in children with acute viral infections [30, 31]. Viral immune activation may transiently enhance T‐cell reactivity to drugs, leading to delayed maculopapular eruptions that often do not reflect persistent allergies. Accordingly, many children tolerate aminopenicillins and have negative allergy testing results in later evaluations, including PTs and drug provocation tests [30, 31].

Overall, the current paediatric data indicate that PT has limited standalone diagnostic value in common non‐immediate beta‐lactam reactions such as maculopapular exanthema. Its role appears more complementary than decisive and is potentially useful in selected severe phenotypes or when intradermal testing is contraindicated or unavailable, as certain beta‐lactam antibiotics are not commercially available in sterile formulations suitable for intradermal testing. However, it is insufficient to replace drug provocation testing when clinically feasible.

### 3.2 Non‐Beta‐Lactam Antibiotics

Evidence regarding the role of PT for non‐beta‐lactam antibiotics in children remains limited and is largely derived from small retrospective cohorts and isolated case reports, which limit the ability to draw robust conclusions from the data. However, in the available paediatric series, PT positivity appears to be uncommon.

In a cohort in which 10/45 antibiotic PTs involved non‐beta‐lactam agents with a history of mild reactions (including macrolides, quinolones, trimethoprim–sulfamethoxazole, teicoplanin, clindamycin, and ornidazole), all tests were negative [17]. Similarly, in paediatric patients with SCARs, PT results have been inconsistent but generally limited. In DRESS, positivity was reported in one case (e.g., clindamycin), whereas in SJS/TEN, all PTs performed with non‐beta‐lactam antibiotics were negative [4]. In another retrospective cohort study in which non‐beta‐lactam antibiotics accounted for a minority of suspected cases, PT positivity was rare, with isolated reports involving vancomycin in maculopapular exanthema. Notably, despite being implicated in several suspected clinical reactions, fluoroquinolones consistently yielded negative PT results [26].

Finally, a case report described a 17‐year‐old patient with multidrug‐resistant tuberculosis who developed anaphylaxis 24 h after the initiation of second‐line antituberculosis therapy [32]. PT was strongly positive for clofazimine, moderately positive for kanamycin, and mildly positive for cycloserine, whereas it tested negative for moxifloxacin, amoxicillin–clavulanate, and cotrimoxazole. Clofazimine was discontinued, and graded rechallenge with kanamycin and cycloserine was successfully performed without recurrence of symptoms.

Overall, the current paediatric literature suggests that PT has a limited and largely adjunctive role in evaluating non‐beta‐lactam antibiotic hypersensitivity. However, the data are heterogeneous, and most of them arise from small or highly selected populations.

### 3.3 NSAIDs and Paracetamol

Paracetamol and NSAIDs are the most frequently used analgesic and antipyretic agents in paediatric medicine, with corresponding potential for hypersensitivity reactions. While immediate‐type reactions (urticaria, angioedema, and anaphylaxis) are well recognized, DHRs to these agents are considerably less common but well documented in adult and paediatric populations [33, 34]. Paediatric cases of delayed hypersensitivity to paracetamol confirmed by PT included a 2‐year‐old boy who developed a delayed systemic reaction to paracetamol, which was subsequently confirmed by positive PT and oral provocation [35]. Additionally, a 5‐year‐old child with AGEP following paracetamol ingestion has been reported [36].

Published evidence on PT with NSAIDs in paediatric populations is similarly limited, with most NSAID‐induced delayed hypersensitivity reactions in children being evaluated primarily through oral provocation testing rather than PT [37]. This is due to the considerable variability in NSAID structures and the possibility of cross‐reactivity patterns, which necessitate comprehensive testing with multiple NSAID representatives if PT is performed [38]. In a retrospective observational study including 54 paediatric patients with SCARs, three PTs were performed in three cases of SJS/TEN (testing ketoprofen, acetylsalicylic acid, and ibuprofen), and all yielded negative results [4].

Overall, as observed for other drug classes, the role of PT in the evaluation of DHRs to paracetamol and NSAIDs in children appears to be limited, with the available evidence largely derived from isolated case reports and small observational series. The scarcity of paediatric‐specific studies and the heterogeneity of reported cases currently preclude a clear definition of the diagnostic value of PT for these agents in routine clinical practice.

### 3.4 AEDs and Neuroleptics

PTs are an important diagnostic approach for evaluating delayed hypersensitivity reactions to AEDs and, to a lesser extent, neuroleptic agents. In paediatric patients with suspected anticonvulsant hypersensitivity, PTs provide a relatively safe and informative method for identifying the causative drug, evaluating potential cross‐reactivity, and guiding the selection of alternative therapies [17]. This is particularly relevant in SCARs such as DRESS and SJS/TEN, in which drug provocation tests are associated with the risk of life‐threatening recurrences. PTs are especially valuable when several medications are administered concurrently, as they may help identify the specific AED responsible for the reaction [39, 40]. Additionally, it can help assess the cross‐reactivity among structurally related compounds. Aromatic AEDs (e.g., carbamazepine, phenobarbital, and phenytoin) are well known for their high rate of cross‐reactivity, reported in approximately 40%–80% of cases [41]. Therefore, PT can support clinical decision‐making when switching to alternative agents such as non‐aromatic AEDs (e.g., levetiracetam) [42].

Evidence from paediatric studies and case series supports the clinical utility of PT for AED hypersensitivity. In a cohort of 71 children with mild or moderate reactions and SCARs undergoing drug PT, approximately 28% of the tests were positive overall, with a significantly higher positivity rate for AEDs (40.9%) than for antibiotics (4.1%) [17]. In another paediatric series, positive PT results were observed in 59% of children with suspected hypersensitivity to AEDs [43]. Among the individual agents, carbamazepine consistently demonstrated the highest diagnostic yield, with a reported positivity rate of approximately 63%–66% [17]. Positive responses have been reported for other AEDs, including phenobarbital (approximately 33%–86%), lamotrigine (approximately 41%), and valproic acid (approximately 27%–63%) [43, 44, 45]. Although rare, PTs positive for ethosuximide have been described, including cases of SJS/TEN overlap in children [46].

### 3.5 Other Drugs

PT can provide information about the causative role of drugs in rare or previously unreported delayed hypersensitivity reactions. One illustrative case involved a 16‐year‐old patient who developed a lichenoid drug eruption approximately 2 months after a single vaginal administration of misoprostol. PT performed with 1% misoprostol in petrolatum elicited a strong positive (++) reaction at days 2 and 7, confirming that the drug was the causative agent and representing the first reported case of misoprostol‐induced lichenoid eruption confirmed by PT [47].

In a reported case involving a 6‐year‐old boy who developed a generalized pustular eruption after treatment with oral terbinafine for tinea capitis, PT induced a localized pustular reaction at the terbinafine test site while remaining negative for amoxicillin–clavulanic acid, which had also been administered. These findings confirmed that terbinafine was the causative agent and represented only the second paediatric case of terbinafine‐induced AGEP confirmed through PT [48]. Similarly, PT has been used to investigate hypersensitivity reactions to inhaled corticosteroids. Two patients who presented with respiratory and cutaneous symptoms (including angioedema, nasal congestion, and pruritus) after the use of intranasal or inhaled budesonide showed strong positive (++) PT reactions to budesonide [49]. In one case, cross‐reactivity to tixocortol‐21‐pivalate was observed, underscoring the importance of testing structurally related corticosteroids to guide the selection of safe alternative treatments.

In a retrospective observational study of 54 paediatric patients with SCARs, PT was performed in cases of AGEP; one PT for hydroxychloroquine yielded a positive result, whereas one PT for terbinafine was negative [4].

## 4 Conclusion

**FIGURE 1:**
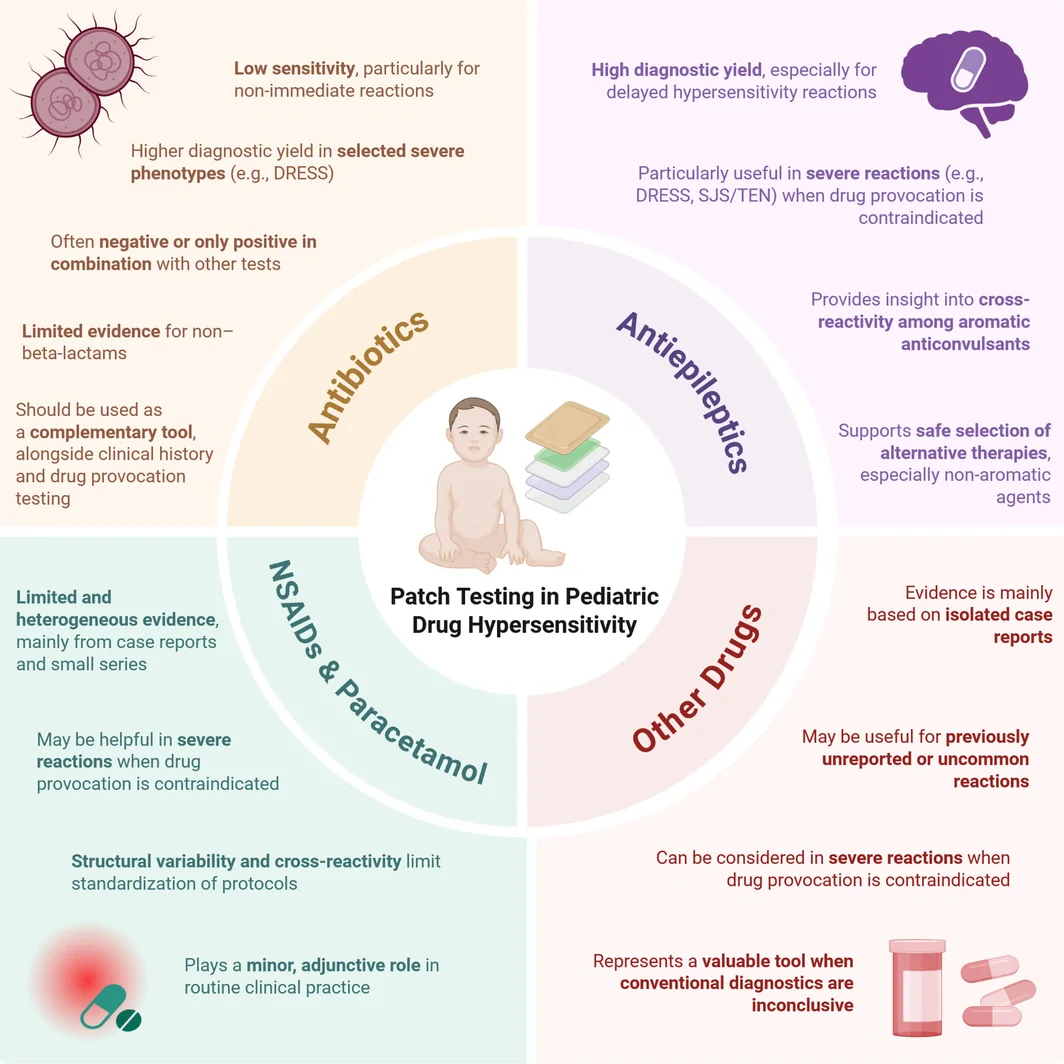
Diagnostic role of patch testing in paediatric drug hypersensitivity across drug classes.

PT is a valuable diagnostic tool for evaluating delayed DHRs in the paediatric population. In children, where drug provocation testing may be contraindicated or associated with a significant risk, particularly in SCARs, PTs provide an important non‐invasive complementary approach within the diagnostic work‐up (Figure 1). However, the diagnostic performance of PT varies substantially depending on the drug class and the clinical phenotype of the reaction. Available paediatric data indicate that the sensitivity of PT is generally low for beta‐lactam antibiotics and most non–beta‐lactam antibiotics, limiting its role as a standalone diagnostic method in these contexts. In contrast, higher diagnostic yields have been reported for AEDs, particularly aromatic AEDs such as carbamazepine, phenobarbital, and phenytoin, where PT may help confirm the causative agent and assess potential cross‐reactivity among structurally related compounds. Evidence for NSAIDs and paracetamol remains limited, whereas isolated case reports have demonstrated that PT can also help confirm hypersensitivity to less commonly implicated drugs.

Despite these potential applications, several limitations affect the clinical utility of PT for paediatric drug allergies. Methodological heterogeneity, including variability in drug concentrations, vehicles, preparation methods, and interpretation of results, remains a major challenge. Additionally, paediatric‐specific data for many drug classes remain scarce, and the lack of standardized protocols may contribute to the variability in reported sensitivities and diagnostic yields across studies and centres. Consequently, PT should always be interpreted within a broader clinical context, integrating the patient's history, reaction phenotype, and the results of other diagnostic procedures.

To the best of our knowledge, this work represents the first informative and descriptive review specifically addressing the diagnostic performance of PT for DHRs in the paediatric population, providing a comprehensive synthesis of the currently available evidence in this field. Future research should focus on the development of standardized paediatric protocols, the validation of optimal drug concentrations and vehicles, and prospective multicentre studies aimed at better defining the diagnostic accuracy of PT across different drug classes and reaction phenotypes. Improving methodological consistency and expanding paediatric‐specific evidence are essential to clarify the role of PT in the diagnostic algorithms of paediatric DHRs. Ultimately, more precise and evidence‐based use of PT may contribute to improving drug allergy diagnosis, reducing unnecessary drug avoidance, and supporting safer therapeutic choices in the paediatric population.

## Author Contributions

**Gabriele Simonetti:** conceptualization, writing – original draft, writing – review and editing, data curation. **Leonardo Tomei:** writing – original draft, supervision. **Francesca Mori:** writing – original draft, conceptualization, validation, methodology, supervision. **Giulia Liccioli:** writing – original draft, supervision. **Lucrezia Sarti:** writing – original draft, supervision. **Simona Barni:** writing – original draft, supervision, visualization. **Francesco Catamerò:** conceptualization, investigation, writing – original draft, methodology, writing – review and editing, supervision. **Mattia Giovannini:** writing – original draft, supervision, resources.

## Funding

The authors have nothing to report.

## Ethics Statement

The authors have nothing to report.

## Conflicts of Interest

F.C. reports personal fees from Hippo Dx. S.B. reports personal fees from Sanofi and Firma. M.G. reports personal fees from Sanofi and Thermo Fisher Scientific. All other authors report no conflicts of interest related to this work.

## Data Availability

The data that support the findings of this study are available from the corresponding author upon reasonable request.
